# Supplementary material for: Spatial neglect after subcortical stroke may reflect cortico-cortical disconnection
Source: Sci Rep. 2025 May 27;15:18544. doi: 10.1038/s41598-025-01703-x (PMC12117021; doi:10.1038/s41598-025-01703-x)
Supplement: Supplementary file 1 — Supplementary Material 1 [file 41598_2025_1703_MOESM1_ESM.pdf]

# Supplementary

## Spatial neglect after subcortical stroke: sometimes a cortico-cortical disconnection syndrome

Christoph Sperber, Hannah Rosenzopf, Max Wawrzyniak, Julian Klingbeil, Dorothee Saur, Hans-Otto Karnath

***Lesion-network mapping – detailed procedures***

Reference rs-fMRI data were taken from the human connectome project (van Essen et al., 2013) and included two resting-state sessions (with right-left and left-right phase encoding) of 15 minutes acquired with a gradient-echo echo planar imaging sequence (TR = 720 ms, 2 x 2 x 2 mm isotropic resolution). All images were already 'minimally preprocessed' when downloaded. This included gradient distortion correction, motion correction, distortion correction, normalization to MNI space, intensity normalization and bias field removal (Glasser et al., 2013). Functional images were smoothed (FWHM 5mm) and linearly corrected for motion parameters and their first derivate, and for mean white matter, cerebrospinal fluid and global signal (defined by tissue probability > 0.95). The remaining signal was band-pass filtered to retain frequencies between 0.01 and 0.08 Hz (Fox and Raichle, 2007). Motion Scrubbing was applied by calculating frame-wise displacement (Power et al., 2012) and discarding volumes with a value > 0.5mm. Two datasets were excluded because of heavy in-scanner motion with < 10 minutes of rs-fMRI after motion scrubbing in at least one session.

For the next level analyses, we used the binary normalized lesion map of each patient as a region of interest. Only grey matter portions of lesion maps were used in this step to ensure that included areas are subject to a meaningful BOLD signal. To this end, we utilised the grey matter tissue probability mask shipped with SPM12 at a probability threshold of > 0.1. Functional connectivity was computed as the Fisher-transformed Pearson correlation between the time series' first eigenvariate within the seed region and the time series of all remaining voxels. We calculated separate connectivity maps for the left-right and right-left phase encoding session. This procedure was repeated for each healthy subject and resulting topographies were averaged to produce a single topography ('lesion-network') per patient.

We utilised patient-wise maps of averaged Fisher-transformed Pearson correlations as lesion-network maps. For statistical analyses, the full-brain lesion-network maps have been masked to only include cerebral cortical grey matter areas within the AICHA brain atlas (Joliot et al., 2015), termed cortical lesion-network maps.

***Supplementary References***

Fox, M.D., Raichle, M.E., 2007. Spontaneous fluctuations in brain activity observed with functional magnetic resonance imaging. Nat Rev Neurosci 8, 700–711. https://doi.org/nrn2201 [pii]\n10.1038/nrn2201

Glasser, M.F., Sotiropoulos, S.N., Wilson, J.A., Coalson, T.S., Fischl, B., Andersson, J.L., Xu, J., Jbabdi, S., Webster, M., Polimeni, J.R., Van Essen, D.C., Jenkinson, M., 2013. The minimal preprocessing pipelines for the Human Connectome Project. Neuroimage 80, 105–124. https://doi.org/10.1016/j.neuroimage.2013.04.127

Joliot, M., Jobard, G., Naveau, M., Delcroix, N., Petit, L., Zago, L., Crivello, F., Mellet, E., Mazoyer, B., Tzourio-Mazoyer, N., 2015. AICHA: An atlas of intrinsic connectivity of homotopic areas. J. Neurosci. Methods 254, 46–59. https://doi.org/10.1016/j.jneumeth.2015.07.013

Power, J.D., Barnes, K.A., Snyder, A.Z., Schlaggar, B.L., Petersen, S.E., 2012. Spurious but systematic correlations in functional connectivity MRI networks arise from subject motion. Neuroimage 59, 2142–2154. https://doi.org/10.1016/j.neuroimage.2011.10.018

Van Essen, D.C., Smith, S.M., Barch, D.M., Behrens, T.E.J., Yacoub, E., Ugurbil, K., 2013. The WU-Minn Human Connectome Project: An overview. Neuroimage 80, 62–79. https://doi.org/10.1016/j.neuroimage.2013.05.041

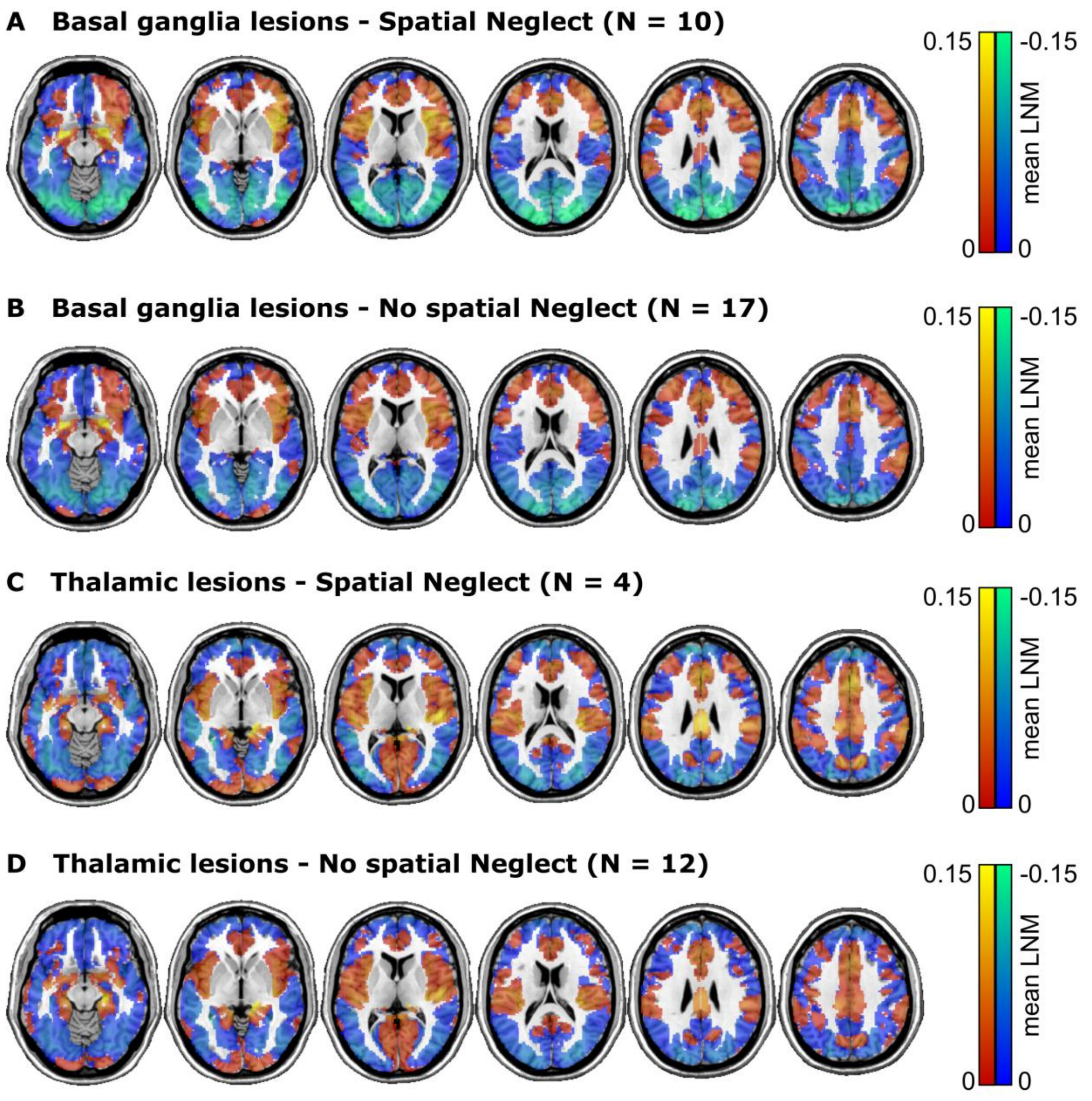


**Supplementary Figure 1**: Mean Lesion Network connectivity (i.e. correlation scores) across all patients per group. Values may focally exceed the scaling threshold of 0.15/-0.15, which have been chosen to optimise the visualisation of data in smaller ranges.

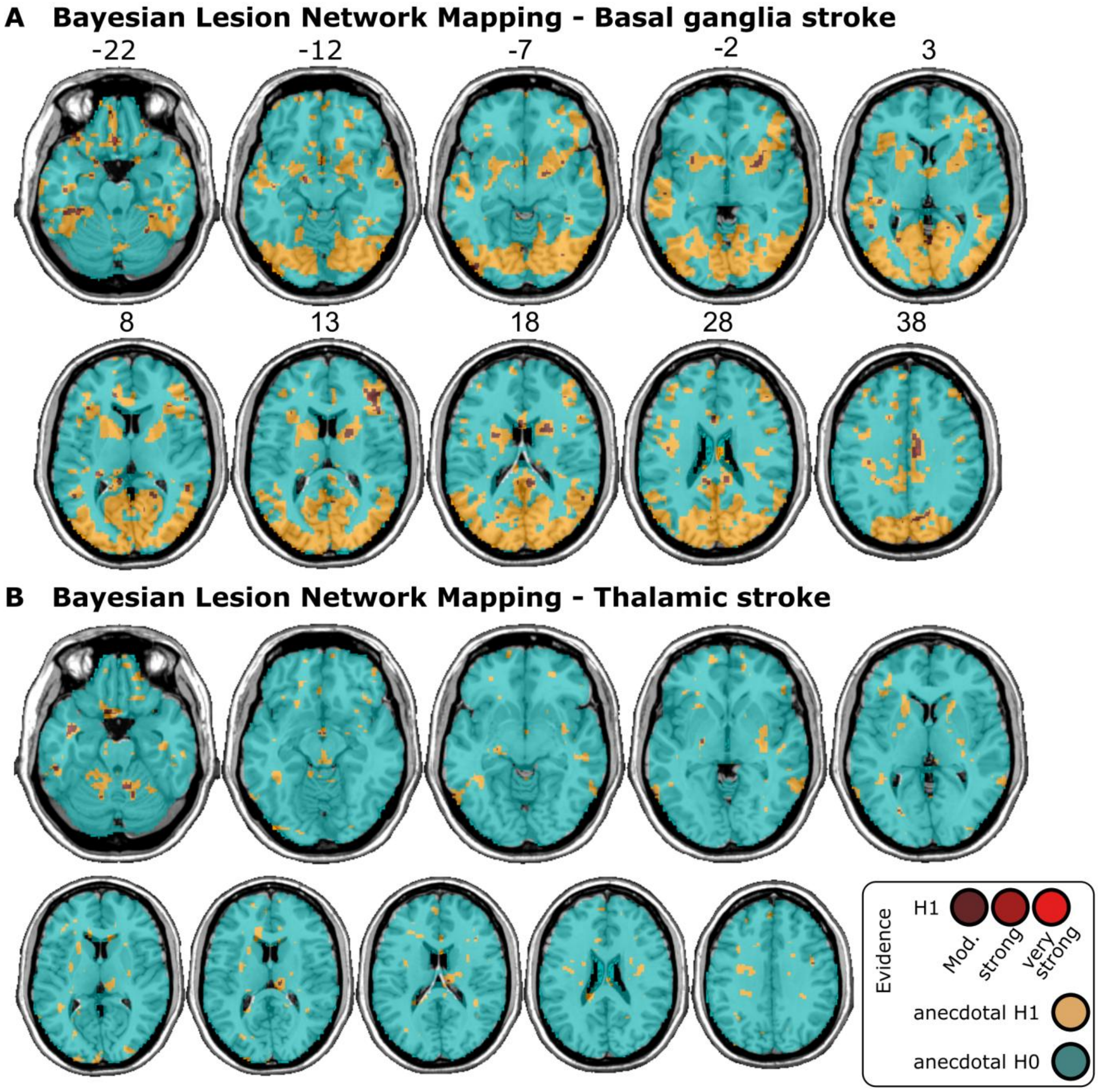


**Supplementary Figure 2**: Un-masked results of the Bayesian lesion-network mapping analysis, including also signal in the cerebral white matter and the basal ganglia. Note that results in the right basal ganglia can be explained by autocorrelation, i.e. correlation of fMRI signal in the lesion area with itself. **(A)** Statistical results of the Bayesian voxel-wise lesion-network analysis with normative rsfMRI data for basal ganglia stroke and **(B)** thalamic stroke. The analyses resulted in Bayes factors that weigh the evidence for the alternative hypothesis $h_1$ (i.e. an association between the voxel-wise lesion-network connectivity and spatial neglect exists) and the null hypothesis $h_0$ (i.e. no such association exists). For visualisation, we binned these Bayes factors according to common conventions (Wagenmakers et al., 2018) into evidence in favour of $h_1$ (red; moderate BF > 3, strong BF > 10, very strong BF > 30), and inconclusive, anecdotal evidence for h1 (yellow; BF > 1) anecdotal evidence for h0 (turquoise; BF < 1). Due to the small sample sizes, the smallest Bayes factor was 0.35 (i.e. > 1/3), hence no moderate or stronger evidence for h0 was found.

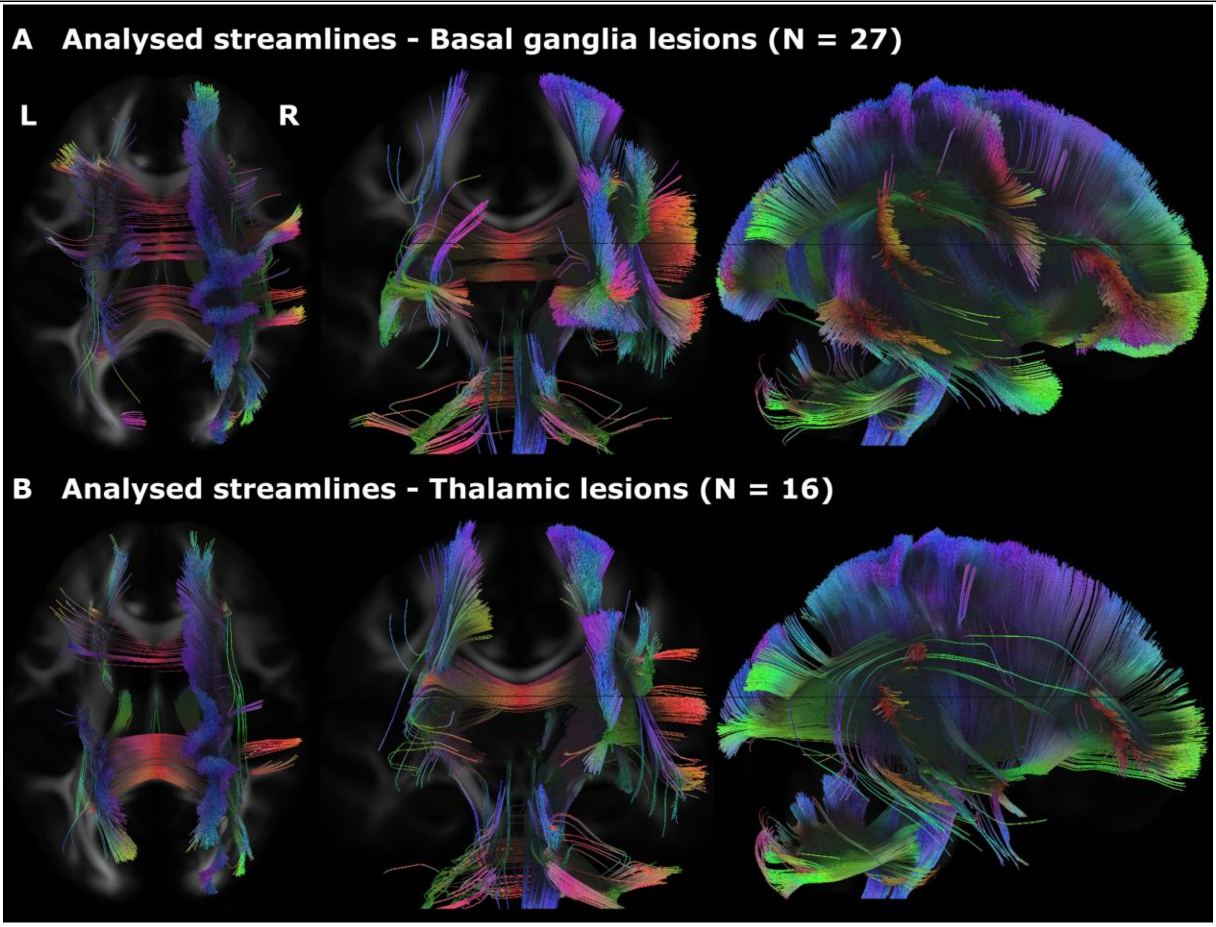


**Supplementary Figure 3**: Streamlines that were disconnected in at least 5 patients and hence were statistically analysed in Experiment 2. Colours code the directionality of streamlines.
